# Genetic Diversity and Population Structure of *Metaphire vulgaris* Based on the Mitochondrial COI Gene and Microsatellites

**DOI:** 10.3389/fgene.2021.686246

**Published:** 2021-06-08

**Authors:** Yu Fang, Jie Chen, Honghua Ruan, Nan Xu, Ziting Que, Hongyi Liu

**Affiliations:** ^1^College of Biology and the Environment, Nanjing Forestry University, Nanjing, China; ^2^Key Laboratory for Ecology and Pollution Control of Coastal Wetlands (Environmental Protection, Department of Jiangsu), School of Environmental Science and Engineering, Yancheng Institute of Technology, Yancheng, China

**Keywords:** COI, microsatellite, genetic diversity, *Metaphire guillelmi*, *Metaphire vulgaris*

## Abstract

The earthworm species *Metaphire vulgaris* (a member of the Clitellata class) is widely distributed across China, and has important ecological functions and medicinal value. However, investigations into its genetic diversity and differentiation are scarce. Consequently, we evaluated the genetic diversity of five populations of *M. vulgaris* (GM, HD, NYYZ, QDDY, and QDY) in Yancheng, China via the mitochondrial COI gene and the novel microsatellites developed there. A total of nine haplotypes were obtained by sequencing the mitochondrial COI gene, among which NYYZ and QDDY populations had the greatest number of haplotypes (nh = 5). Further, the nucleotide diversity ranged from 0.00437 to 0.1243. The neighbor-joining trees and the TCS network of haplotypes indicated that earthworm populations within close geographical range were not genetically isolated at these small scale distances. Results of the identification of microsatellite molecular markers revealed that the allele number in 12 microsatellite loci ranged from 4 to 13. The observed heterozygosity ranged from 0.151 to 0.644, whereas the expected heterozygosity ranged from 0.213 to 0.847. The polymorphism data content of most sites was >0.5, which indicated that the designed sites had high polymorphism. Structural analysis results indicated that GM, HD, and NYYZ had similar genetic structures across the five populations. The Nei’s genetic distance between HD and NYYZ populations was the smallest (*D*_*s*_ = 0.0624), whereas that between HD and QDY populations was the largest (*D*_*s*_ = 0.2364). The UPGMA tree showed that HD were initially grouped with NYYZ, followed by GM, and then with QDDY. Furthermore, cross-species amplification tests were conducted for *Metaphire guillelmi*, which indicated that the presented markers were usable for this species. This study comprised a preliminary study on the genetic diversity of *M. vulgaris*, which provides basic data for future investigations into this species.

## Introduction

Earthworms have key roles in myriad soil processes, including soil turnover, aeration, and drainage, and the breakdown and incorporation of organic matter (Edwards and Bohlen, 1996). Studies have revealed that direct interactions between earthworms and seeds can influence the formation of plant communities (Asshoff et al., 2010). Against the backdrop of escalating terrestrial pollution, earthworms can accelerate the degradation of soil permeating pesticide residues (Liang and Zhou, 2006; Lin et al., 2018). Furthermore, from a medical perspective, earthworms can be employed for the prevention and cure of arteriosclerosis, promotion of blood circulation, and removal of blood stasis, as well as the prevention and treatment of cardiovascular and cerebrovascular diseases. Thus, it is important to elucidate the diversity and population structures of earthworms. Traditional morphological studies begin with phenotypes; however, phenotypes are generally controlled by genes and are significantly influenced by the environment (Gilbert and Nijland, 2008). Therefore, it is difficult to accurately determine the level of genetic variation between species through phenotypic differences.

At present, mitochondrial DNA (mtDNA) and microsatellites are extensively employed for species identification, population genetic diversity, and genetic differentiation (Guichoux et al., 2011; Hodel et al., 2016; Qin et al., 2017). mtDNA is a type of extranuclear genetic material (Herrera et al., 2015). Some authors have analyzed mtDNA to investigate the genetic diversity and population structures of earthworms (Chang and Chen, 2005; Minamiya et al., 2009; Lang et al., 2012; Siqueira et al., 2013). Mitochondrial COI genes are also the most commonly used molecular markers. Molecular genetic studies have demonstrated that the relative paucity in morphological characteristics conceals a high genetic diversity (Shekhovtsov et al., 2014). Microsatellites are simple repeat sequences with 1–6 bases as the repeating unit (Babaei et al., 2017), which have been confirmed to be very suitable markers for the study of population genetics. However, cross-species amplification experiments have revealed that earthworm microsatellite marker possess a high specificity for species (Guichoux et al., 2011). Only a few sets of microsatellite markers of Megascolecidae earthworms have been developed (e.g., *Amynthas corticis*) (Cunha et al., 2017). Further, studies on the genetic diversity of earthworms via microsatellites have been mostly for the Lumbricidae family (Somer et al., 2011; Dupont et al., 2017).

In response to the growing need for genetic and genomic tools for the study of earthworm biology, we isolated 12 microsatellite markers for *Metaphire vulgaris* using RAD sequencing technologies. *M. vulgaris* belongs to the genus *Metaphire* of the family Megascolecidae, which can be found in many provinces across China, including Jiangsu, Shanghai, Zhejiang, and Guizhou (Xu and Xiao, 2011). To provide theoretical data for the level of genetic diversity of this species, which belonged to different ecosystems in Yancheng City of Jiangsu Province, the genetic diversity and population structures were evaluated using mitochondrial COI gene and the novel microsatellites. These data can contribute to elucidating the genetic diversity and differentiation of the *M. vulgaris* group of earthworms, as well myriad other species.

## Materials and Methods

### Sample Collection and DNA Extraction

**FIGURE 1:**
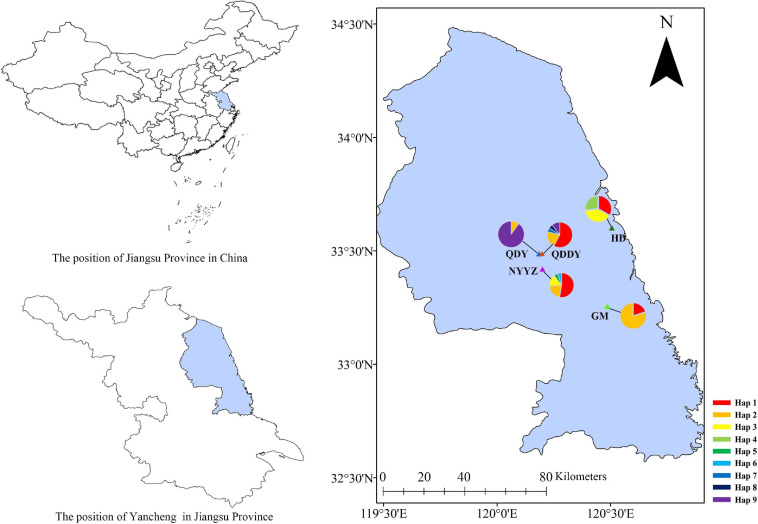
Distribution of sampling locations and haplotypes.

All earthworm samples were selected from five sites in Yancheng City, Jiangsu Province, China and grouped according to their geographic origin (Figure 1 and Table 1). A total of 112 earthworms were collected as follows: 15 earthworms from Guomeng Town (GM) (120°28’41.4″E, 33°15′23.6″N), 30 from Seawall Road (HD) (120°30’24.8″E, 33°36’2.5″N), 21 from rape and pea fields in Qingdun Town (QDDY) (120°11’11.3″E, 33°29’14.9″N), 21 from a rape field in Qingdun Town (QDY) (120°11’55.5″E, 33°29’18.5″N), and 25 from the Nanyang Experimental Station (NYYZ) (120°12’5.1″E, 33°25′13.1″N). GM and QDDY reside in long-term cultivated lands (GM was sampled on the ridge of the field), whereas HD and NYYZ dwell in agricultural wastelands, and QDY is present in newly cultivated land. The earthworms were anaesthetized in the field with 10% ethanol, and subsequently preserved in 70% ethanol. The *M. vulgaris* were from 130 to 150 mm in length and 5–7 mm wide. The body surface has no setae, and the color of the middle line on the back is dark cyan. The mating cavity is deep and wide, and the inner wall is wrinkled, often with three flat-topped mastoid processes. The anterior and posterior margin of the seminal vesicle is swollen, and the size of the mastoid process can be seen outside the lumen (Xu and Xiao, 2011). Once the earthworms were identified with similar *M. vulgaris*, samples from each individual were sectioned and preserved in 95% ethanol for genomic DNA extraction using a genomic DNA extraction kit (Vazyme Biotech, Beijing, China) in the laboratory. The extracted DNA was stored at −20°C for an extended duration.

**TABLE 1:** Geographic locations of sampling sites and haplotypes.

**Species**	**ID name**	**Site**	***N***	**π**	**nh**	**Hd**	***S***
*Metaphire vulgaris*	GM	120°28′41.4″E, 33°15′23.6″N	5	0.00868 ± 0.01042	2	0.400 ± 0.05632	16
	HD	120°30′24.8″E, 33°36′2.5″N	15	0.01243 ± 0.00751	3	0.705 ± 0.00286	18
	NYYZ	120°12′5.1″E, 33°25′13.1″N	19	0.01184 ± 0.00738	5	0.684 ± 0.00841	19
	QDDY	120°11′11.3″E, 33°29′14.9″N	19	0.01060 ± 0.00776	5	0.637 ± 0.01093	20
	QDY	120°11′55.5″E, 33°29′18.5″N	20	0.00437 ± 0.00650	2	0.189 ± 0.01169	17
All *M. vulgaris*			78	0.01088 ± 0.00633	9	0.776 ± 0.00061	23
All *M. guillelmi*			22	0.00000 ± 0.00000	1	0.000 ± 0.00000	0
Overall			100	0.02646 ± 0.01441	10	0.817 ± 0.00024	55

*N, number of sequences; π, nucleotide diversity; nh, number of haplotypes; Hd, haplotype diversity; S, number of segregation sites.*

### COI Gene Amplification and Molecular Identification of Species

The primers used for gene amplification were designed with reference to the entire mtDNA sequences of *M. vulgaris* (KJ137279.1), *Metaphire guillelmi* (KT429017.1), and *Metaphire californica* (KP688581.1) from NCBI (QY-COI-F:5′-TTTGGGCACCCAGAAGTATA-3′; QY-COI-R:5′-GTAATAATACCTGTTTCYCT-3′). Amplifications were performed in 25 μl reaction volumes containing 12.5 μl of 2 × Taq Master Mix (Dye Plus), 1 μl of genomic DNA, 0.5 μl of each primer, and 10.5 μl of deionized water. The PCR procedure was as follows: an initial denaturation step at 95°C for 5 min, followed by 32 cycles of denaturation at 95°C for 30 s, annealing at 55°C for 30 s, and extension at 72°C for 30 s, with a final extension at 72°C for 5 min. After being detected by agarose gel electrophoresis, the PCR products were sent to the TSINGKE Biotech Company (Nanjing, China) for sequencing. Species identification was performed via DNA barcoding by sequencing a fragment of the COI gene. Ultimately, we obtained 78 *M. vulgaris* and 22 *M. guillelmi* earthworms.

### Microsatellite Identification and Amplification

The genomic DNA of a *M. vulgaris* specimen was used for RAD-seq, where RAD library construction and Illumina sequencing were conducted by Novogene Bioinformatics Technology Co. Ltd. (Beijing, China) following the standard protocol. Approximately 1.287 G bases of raw reads were obtained from the RAD library, with average Q30 and GC contents of 92.41 and 43.51%, respectively. Subsequent to the filtering and assembly of the raw reads, we obtained 33,069 contigs, with average contig lengths of 346 bp. A total of 1975 microsatellites were obtained that were suitable for the design of primers. The primers were designed using the primer 3.0 subprogram of the SR search software (Novogene, Beijing, China). A total of 20 pairs of primers were randomly designed and employed to amplify the DNA templates of 3 *M. vulgaris* individuals, of which 12 pairs of primers produced clean products. These primers were labeled with fluorescent dye 5′ 6-FAM, 5′ HEX, or 5′ TAMRA for randomly testing the amplification in 24 *M. vulgaris* individuals. Finally, 12 microsatellite loci with high polymorphisms and 12 corresponding primers were successfully screened (Table 2).

**TABLE 2:** Characteristics of the 12 microsatellite primers.

**Locus**	**Primer sequences(5′–3′)**	**Repeat type**	**Fluorescent markers**	***T*_*m*_/°C**
Mv01	F:GTTTTGAAATTATCTGTCG	(CA)_9_	HEX	55
	R:TCTCGCCACTTTTATCACAC			
Mv02	F:ATTATTTTGACGCTTCCATAC	(GT)_7_	HEX	55
	R:GTTCCTTTGATCTCTCGTAA			
Mv03	F:TGGAGCTCAGTCTGTCTGTC	(CTGT)_7_	HEX	55
	R:TGAACCCTTCTCTCTACCCC			
Mv04	F:TCCCAAGAGTATTGAGGATTT	(CT)_15_	TAMRA	55
	R:ACTAGCATAGCGTGTGCGTG			
Mv05	F:TAAACTTCGACCCACACTGA	(CAG)_4_	TAMRA	55
	R:CGTCTGACCTAAGAAGTCCC			
Mv06	F:ATATGGTTGCAAAAACAATCA	(GT)_11_	TAMRA	55
	R:GTTGTGCATTCCTGTTTAGAA			
Mv07	F:CATAATTAGCTCCACTCGG	(AG)_15_	HEX	55
	R:GTTGTGCATTCCTGTTTAGAA			
Mv08	F:GAAATGAAGCTGAGATGACA	(CTCA)_9_	TAMRA	55
	R:TGGAACGAAACATAGAGGG			
Mv09	F:TGAGGACTGGTTTGACACTT	(CTG)_6_	FAM	55
	R:TAACCAGTTCCGTTTGCTCTC			
Mv10	F:AGGTCAGCATCGACGACGACAAC	(CCG)_5_	FAM	55
	R:CCTTTCCACCACCCTATCGT			
Mv11	F:AGGAGGAGATGAAAATATCG	(GAGG)_5_	FAM	55
	R:AGCACCAAAGATGAGATGGA			
Mv12	F:CGACGTCCATCTACTTTGAA	(TG)_16_	FAM	55
	R:CAAAAATAGTTTGACAAGCA			

Except for different primers, the reaction system and reaction conditions were as above. The PCR products were also checked using a 1% agarose gel electrophoresis method. To validate the developed microsatellites in other *Metaphire* species, *M. guillelmi* (*n* = 22) were sampled for cross-amplification analysis. All PCR products were sent to the SINGKE Biotech Company (Nanjing, China) for genotyping using an ANI 3730 Genetic Analyzer (Applied Biosystem).

### Statistical Analyses

#### mtDNA Sequence Data

The sequences of each gene region were edited and aligned in SeqMan (Swindell and Plasterer, 1997) Pro v9 (DNAstar Inc., Madison, WI, United States). Molecular genetic diversity indices for each population were calculated in DnaSP v5.0 (Librado and Rozas, 2009). The diversity indices included nucleotide diversity (π), number of haplotypes (nh), haplotype diversity (Hd), and number of segregation sites (S). A neighbor joining (NJ) tree was constructed by MEGA v7.0 (Sudhir et al., 2016) according to the haplotype of the population, and with *M. guillelmi* as an outgroup. The confidence levels at nodes after 1000 repetitions employed the Bootstrap method (Adeniran et al., 2021). The phylogenetic relationships between mtDNA haplotypes of *M. vulgaris* were estimated from a TCS network using PopART v1.7 (Leigh and Bryant, 2015).

#### Microsatellite Data

Cervus version 3.0 software (Kalinowski et al., 2007) was used to determine the following parameters: The number of alleles (N_*A*_), observed heterozygosity (H_*O*_), expected heterozygosity (H_*E*_), polymorphism information content (PIC) values, and Hardy–Weinberg equilibrium test for each locus. Arlequin 3.0 software (Excoffier et al., 2007) was employed to estimate the fixation indices (F_*IS*_, F_*ST*_, and F_*IT*_) per locus. Through an AMOVA analysis using Arlequin 3.0 software, the distribution patterns of genetic diversity were compared. Popgene 3.2 software was employed to calculate the genetic distance (*D*_*S*_), and construct a phylogenetic tree via UPGMA. An analysis of the population genetic structure was performed with Structure 2.3.4 software (Pritchard et al., 2000), where the Set population *K* 2--5, Each *K* value repeats 10 times, Length of Burnin Period and McMc Reps were 100,000 and 100,000. The results were uploaded to the Structure Harvester^1^ (Rosenberg et al., 2001) to obtain the best *K* value.

## Results

### Population Genetic Diversity and Differentiation of Mitochondrial COI Gene

#### Genetic Diversity of Mitochondrial COI Gene

A total of 100 COI sequences (737 bp) were obtained, of which 78 were *M. vulgaris* and 22 were *M. guillelmi*, following amplification and species identification (GenBank accessions: MW861684–MW861693). The average frequencies of T, C, A, and G were 32.6, 22.3, 27.9, and 17.2%, respectively. The A + T contents (60.5%) were higher than the C + G contents (39.5%). The nucleotide diversity (π), nh, and Hd are presented in Table 1. The NYYZ and QDDY had the most haplotypes (nh = 5). The highest Hd was the HD population (Hd = 0.705), whereas the lowest was the QDY population (Hd = 0.189). The genetic diversity of the GM and QDY populations was significantly lower than that of the other three populations (HD, NYYZ, and QDDY). There were 10 haplotypes in total, among which only one was a *M. guillelmi* haplotype. There were nine haplotypes within the five populations of *M. vulgaris*, among two haplotypes (Hap 1 and Hap 2) were found in four populations, two haplotypes (Hap 3 and Hap 9) were found in two populations, and the remaining five haplotypes were designated as “private haplotypes” (Table 3).

**TABLE 3:** Haplotypes of COI sequences identified in *M. vulgaris* populations.

**Haplotype**	**GM**	**HD**	**NYYZ**	**QDDY**	**QDY**	**Total**	**Relative frequency (%)**
Hap 1	1	5	10	11	0	27	34.62
Hap 2	4	0	4	4	2	14	17.95
Hap 3	0	6	3	0	0	9	11.54
Hap 4	0	4	0	0	0	4	5.13
Hap 5	0	0	1	0	0	1	1.28
Hap 6	0	0	1	0	0	1	1.28
Hap 7	0	0	0	1	0	1	1.28
Hap 8	0	0	0	1	0	1	1.28
Hap 9	0	0	0	2	18	20	25.64

#### Population Genetic Structure of Mitochondrial Gene Markers

**FIGURE 2:**
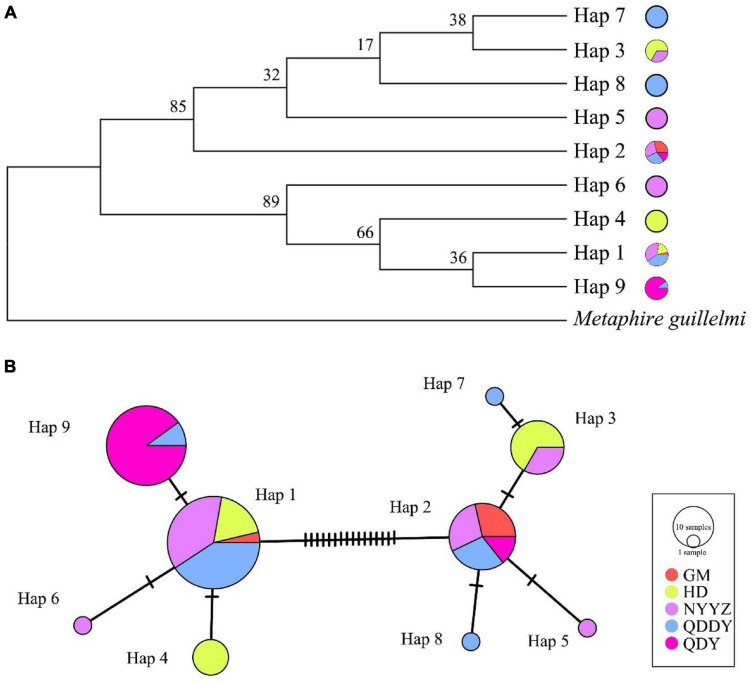
Neighbor-Joining Tree **(A)** and TCS network **(B)** based on COI gene. Circle sizes indicate the probability of haplotypes. Different colored in the circles indicate the distribution in different populations, and the oblique lines indicate mutations between haplotypes.

As was visible from the constructed NJ tree (Figure 2A), the *M. guillelmi* outgroup was obviously different from the *M. vulgaris* group as one branch, and five populations of *M. vulgaris* were divided into two large branches. A total of nine haplotypes were distributed between the two branches, which was similar to the aggregation of the overall TCS network (Figure 2B) haplotype distribution. For most haplotypes, two (Hap 1 and Hap 2) were used as the central radiation distribution. Other haplotypes were formed by one or two mutations of these two haplotypes. Among them, Hap 1 was likely the most primitive haplotype, which evolved into others. The NJ tree and the network between haplotypes revealed that there was no significant lineage differentiation between the five *M. vulgaris* populations.

### Population Genetic Diversity and Structure Based on Microsatellites

#### Genetic Diversity of Microsatellite Loci

An innovative design of the 12 microsatellite loci and their corresponding 12 pairs of primers (GenBank accessions: MW858330–MW858341), a genetic diversity assessment of 73 individuals in 5 populations of *M. vulgaris*, and cross-species amplification of polymorphic microsatellite markers from *M. guillelmi*, showed that most microsatellite loci could be successfully amplified (Table 4). The allele number of all the loci in the 5 different populations of *M. vulgaris* ranged from 4 to 13 with an average number of 8. The H_*O*_ and H_*E*_ ranged from 0.151 to 0.644 (mean value, 0.430), and from 0.213 to 0.847 (mean value, 0.619), respectively. The average PIC was 0.571, while the highest was 0.823 for the Mv07 locus, and the lowest was 0.200 for the Mv08 locus.

**TABLE 4:** Polymorphism of the 12 microsatellite loci for *Metaphire*.

**Locus**	***Metaphire vulgaris***								***Metaphire guillelmi***		
	**N_A_**	**H_O_**	**H_E_**	**PIC**	**HW**	**F_IS_**	**F_ST_**	**F_IT_**	**N_A_**	**H_O_**	**H_E_**
Mv01	13	0.644	0.733	0.739	NS	0.091	0.101	0.183	6	0.471	0.683
Mv02	7	0.507	0.616	0.538	NS	0.086	0.124	0.200	5	0.235	0.652
Mv03	7	0.315	0.608	0.568	*	0.399	0.164	0.498	4	0.235	0.478
Mv04	11	0.548	0.801	0.770	NS	0.267	0.086	0.330	8	0.529	0.736
Mv05	5	0.397	0.579	0.485	NS	0.228	0.141	0.337	7	0.647	0.724
Mv06	4	0.411	0.513	0.440	NS	0.172	0.043	0.208	2	0.529	0.487
Mv07	11	0.575	0.847	0.823	ND	0.317	0.011	0.324	8	0.647	0.838
Mv08	4	0.151	0.213	0.200	ND	0.280	0.027	0.300	5	0.294	0.410
Mv09	7	0.247	0.492	0.448	***	0.466	0.081	0.509	5	0.353	0.711
Mv10	9	0.411	0.621	0.581	NS	0.334	0.012	0.342	6	0.412	0.697
Mv11	5	0.548	0.529	0.452	NS	−0.107	0.080	−0.018	3	0.588	0.594
Mv12	12	0.411	0.836	0.808	ND	0.460	0.114	0.522	5	0.294	0.768
Mean	8	0.430	0.619	0.571	–	0.255	0.085	0.318	5.333	0.436	0.648

N_A_, number of alleles; H_O_, observed heterozygosity; H_E_, expected heterozygosity; PIC, polymorphism information content; F_IS_, fixation index inbreeding coefficient within populations; F_IT_, fixation index inbreeding coefficient in the overall populations; F_ST_, fixation index genetic differentiation; HW, Hardy–Weinberg equilibrium; ND, no deviation from Hardy–Weinberg equilibrium; NS, no significance. *p < 0.05. ***p < 0.01.

#### Population Genetic Diversity and Structure

**FIGURE 3:**
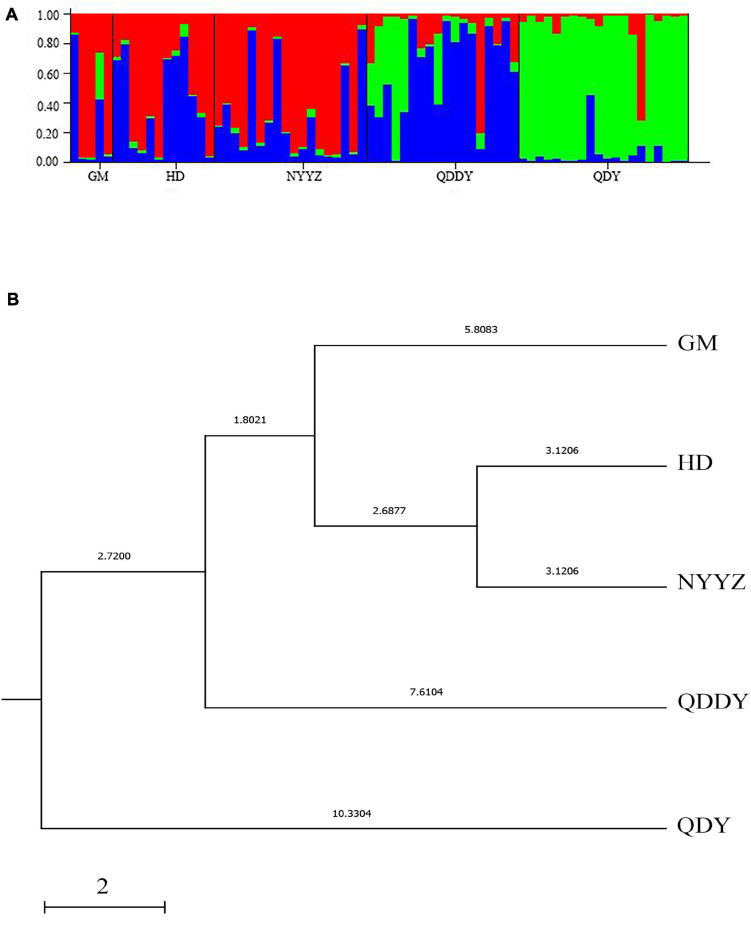
STRUCTURE cluster analysis of the five populations. These populations were grouped in three ancestral clusters **(A)**. The UPGMA tree from 12 microsatellite loci of 5 *M. vulgaris* populations **(B)**.

The N_*A*_ between the five populations of *M. vulgaris* ranged from 3.250 (GM) to 5.250 (QDDY), where the average allele number was 4.42 (Table 5). The ranged from 0.403 (HD) to 0.458 (QDDY). The H_*E*_ ranged from between 0.504 (QDY) and 0.633 (HD). The average of the observed and expected heterozygosity was 0.426 and 0.581, respectively. The PIC was from between 0.446 and 0.546, as the lowest in the QDY population, and the highest in the HD population, with an average of 0.504. The best *K* (*K* = 3) values were obtained from the Structure Harvester (see text footnote 1). The genetic structure of the population was analyzed using Structure 2.3.4 software, setting *K* = 3, that is, the five populations could be divided into three genetic groups (red, blue, and green) (Figure 3A.) All five populations had three simultaneous genetic groups, among which three in the QDDY population were uniformly distributed. The GM, HD, and three NYYZ populations consisted primarily of red and blue-derived genetic populations, whereas QDY were more of the green-derived genetic populations.

**TABLE 5:** Genetic information for the 12 microsatellite loci observed in *M. vulgaris*.

**Population**	**Locus**	**N_A_**	**H_O_**	**H_E_**	**PIC**
GM	Mv01	4	0.400	0.711	0.581
	Mv02	2	0.600	0.467	0.332
	Mv03	2	0.000	0.356	0.269
	Mv04	4	0.600	0.733	0.596
	Mv05	3	0.400	0.689	0.548
	Mv06	2	0.400	0.356	0.269
	Mv07	6	0.600	0.889	0.772
	Mv08	2	0.200	0.200	0.164
	Mv09	3	0.200	0.511	0.410
	Mv10	3	0.200	0.511	0.410
	Mv11	3	1.000	0.733	0.586
	Mv12	5	0.400	0.867	0.745
	Mean	3.250	0.416	0.585	0.473
HD	Mv01	6	0.417	0.717	0.641
	Mv02	4	0.333	0.591	0.501
	Mv03	5	0.333	0.638	0.553
	Mv04	8	0.500	0.801	0.737
	Mv05	3	0.500	0.554	0.428
	Mv06	2	0.333	0.522	0.375
	Mv07	8	0.750	0.855	0.800
	Mv08	2	0.083	0.228	0.195
	Mv09	4	0.417	0.685	0.595
	Mv10	6	0.417	0.710	0.643
	Mv11	4	0.500	0.598	0.483
	Mv12	4	0.250	0.692	0.600
	Mean	4.667	0.403	0.633	0.546
NYYZ	Mv01	6	0.778	0.687	0.625
	Mv02	2	0.500	0.475	0.355
	Mv03	4	0.167	0.257	0.237
	Mv04	8	0.389	0.694	0.635
	Mv05	3	0.611	0.538	0.412
	Mv06	3	0.278	0.510	0.416
	Mv07	11	0.444	0.910	0.873
	Mv08	4	0.278	0.348	0.321
	Mv09	4	0.222	0.611	0.531
	Mv10	4	0.611	0.554	0.494
	Mv11	4	0.444	0.529	0.429
	Mv12	9	0.333	0.816	0.769
	Mean	5.167	0.421	0.577	0.508
QDDY	Mv01	6	0.500	0.675	0.617
	Mv02	6	0.611	0.741	0.679
	Mv03	4	0.556	0.679	0.597
	Mv04	7	0.444	0.808	0.755
	Mv05	3	0.167	0.417	0.370
	Mv06	3	0.389	0.338	0.300
	Mv07	7	0.611	0.830	0.780
	Mv08	2	0.222	0.286	0.239
	Mv09	6	0.389	0.527	0.474
	Mv10	7	0.333	0.665	0.593
	Mv11	4	0.833	0.600	0.504
	Mv12	8	0.444	0.702	0.632
	Mean	5.250	0.458	0.606	0.545
QDY	Mv01	6	0.850	0.754	0.694
	Mv02	4	0.500	0.453	0.406
	Mv03	4	0.300	0.605	0.504
	Mv04	4	0.800	0.688	0.604
	Mv05	4	0.350	0.501	0.438
	Mv06	4	0.600	0.633	0.554
	Mv07	6	0.550	0.738	0.676
	Mv08	1	0.000	0.000	0.000
	Mv09	3	0.050	0.099	0.094
	Mv10	3	0.350	0.573	0.497
	Mv11	2	0.300	0.262	0.222
	Mv12	4	0.550	0.737	0.667
	Mean	3.750	0.433	0.504	0.446

#### Genetic Differentiation of Five Populations

*F*-statistics (Table 4) were estimated in a fixation index as a coefficient within populations (F_*IS*_), genetic differentiation (F_*ST*_), and inbreeding coefficient in the overall populations (F_*IT*_). The F_*IS*_ ranged from −0.107 (Mv11) to 0.466 (Mv09), with an average value of 0.255. The F_*ST*_ ranged from 0.011 (Mv07) to 0.164 (Mv03), with an average value of 0.085. The F_*IT*_ ranged from −0.018 (Mv11) to 0.522 (Mv12), with an average of 0.318. From these three indices, it was observed that there was a certain inbreeding phenomenon; however, the genetic differentiation coefficient was small, which indicated that the degree of genetic differentiation of the population was not high.

According to the allele frequencies of the 5 populations at 12 microsatellite loci, the genetic identity and genetic distance (*D*_*s*_) of Nei was calculated by Popgene v3.2 software. The results indicated that the genetic distance between the HD and NYYZ populations was the smallest (*D*_*s*_ = 0.0624) and the genetic distance between the HD and QDY populations was the largest (*D*_*s*_ = 0.2364) (Table 6). The genetic distances between the GM and HD and NYYZ were also small at 0.1137 and 0.1186, respectively. While the genetic distances between QDY and the other four populations were all larger than 0.16. AMOVA analysis (Table 7) revealed that the total variability observed between different populations was 9.35%, whereas 90.65% of variation was found within populations. The genetic variation of *M. vulgaris* primarily occurred within the population. The UPGMA tree based on codominant genotypic distances matrix of the 12 microsatellite markers from 5 populations showed that HD were initially grouped with NYYZ, followed by GM, and then with QDDY (Figure 3B).

**TABLE 6:** Nei’s genetic identity (above diagonal) and genetic distance (below diagonal) of the five populations of *M. vulgaris* based on microsatellites.

**Population ID**	**GM**	**HD**	**NYYZ**	**QDDY**	**QDY**
GM	****	0.8925	0.8882	0.8578	0.8266
HD	0.1137	****	0.9395	0.8596	0.7895
NYYZ	0.1186	0.0624	****	0.8591	0.8084
QDDY	0.1534	0.1513	0.1519	****	0.8332
QDY	0.1904	0.2364	0.2171	0.1825	****

**TABLE 7:** Analysis of molecular variance for the five populations of *M. vulgaris* based on microsatellites.

**Source of variation**	**d.f.**	**Sum of squares**	**Variance components**	**Percentage variation**
Among populations	4	53.683	0.35418	9.34872
Within populations	141	484.239	3.43432	90.65128
Total	145	537.877	3.78849	

## Discussion

### Amplification of Microsatellite Primers

Microsatellites are markers of neutrality, co-dominance, and high polymorphism. They have been shown to be highly suitable markers for population genetics (Guichoux et al., 2011). However, cross-species amplification tests revealed that the microsatellite markers of earthworms were highly species-specific (*Lumbricus rubellus*, 2006; *Aporrectodea longa* (Ude), 2012; *Lumbricus terrestris*, 2016) (Harper et al., 2006; Strunk et al., 2012; Souleman et al., 2016). At present, there are few studies on the genetic diversity of earthworms using microsatellite molecular markers. For this study, we designed 12 pairs of microsatellite primers for *M. vulgaris*. The PIC values greater than 0.5 for most of the 12 microsatellite loci indicated that these microsatellite markers were highly polymorphic (Yuan et al., 2015). Cross-species amplification tests revealed that the presented markers were usable for *M. guillelmi*. The results of cross-species amplification tests may vary for different families, which can be successfully amplified in Moniligastridae and Megascolecidae, but not in *Lumbricus* (Liu et al., 2020).

### Genetic Diversity of *M. vulgaris* in Yancheng City

Genetic diversity is the foundational core of ecosystems and species diversity, and the basic condition for species to sustain their evolutionary potential (Frankham et al., 2004; Spielman et al., 2004). For mtDNA, the COI gene was used to evaluate the genetic diversity of *M. vulgaris* in Yancheng City. In the present study, the QDY population had the lowest genetic diversity and the HD population had the highest. The COI gene fragment species produced 9 haplotypes in 78 samples of the *M. vulgaris* population with a nucleotide diversity of π = 0.01088 ± 0.00633. This was lower than *Amynthas triastriatus* in China by Dong Yan (π = 0.0309) (Dong et al., 2020), which was primarily related to the small sample size obtained in this study. For the microsatellite makers, 12 microsatellite loci were selected to evaluate the genetic diversity of 73 *M. vulgaris* individuals from the 5 populations in this study. The mean H_*O*_, H_*E*_, and PIC values were 0.430, 0.619, and 0.571 at 12 microsatellite loci, respectively (Table 4). The N_*A*_, H_*E*_, and PIC values of the GM and QDY populations were lower than those of the other populations. The H_*O*_ and the H_*E*_ were 0.426 and 0.581, respectively, based on the microsatellite markers. Compared with *Lumbricus terrestris* (Souleman et al., 2016) (H_*O*_: from 0.132 to 0.839; H_*E*_: from 0.407 to 0.926) studied by Dima Souleman, the population of *M. vulgaris* showed a moderate genetic diversity. The results based on mitochondrial COI gene and microsatellites showed that the genetic diversity of QDY and GM populations was low, whereas that of the HD population was the highest. However, the evaluation of genetic diversity with different molecular markers may give different results (Siqueira et al., 2013). The consistent results of different molecular markers in *M. vulgaris* further indicated the objective existence of genetic diversity in this study (Jiang et al., 2016).

### Population Differentiation and Structure of *M. vulgaris* in Yancheng City

For microsatellites, Nei’s genetic diversity (*D*_*S*_) was calculated to evaluate the level of differentiation between populations. The *D*_*S*_ values between QDY and any other populations was greater than 0.16, which implied that they possessed medium genetic differentiation. The *F*_*ST*_ = 0.09349 (*P* < 0.01) based on microsatellite markers also indicated that genetic differentiation had occurred between the five populations, forming different genetic clusters. According to Bayesian analysis in Structure 2.3.4 software, the five populations of *M. vulgaris* were divided into three genetic clusters. The AMOVA results revealed that the source of genetic differences emerged primarily from within the populations. However, the phylogenetic NJ tree and network based on the species haplotypes of the mitochondrial gene showed no obvious lineage structure. The results of population differentiation based on mitochondrial COI gene and microsatellite molecular markers were inconsistent, which may have been because microsatellites are nuclear genes, while COI are cytoplasmic genes, and the two have different inheritance patterns (Taanman, 1999). In general, earthworms may be considered to be less transmissible animals (Lise et al., 2015) and more likely to form in geographical isolation. However, the results of this study showed that the GM, HD, and NYYZ populations had similar genetic structures, and the three populations were also on a branch in the UPGMA tree. This may have been due to the geographic proximity of the sampling sites and the lack of geographic isolation. Although the geographic locations of the QDDY and QDY populations were similar, the population structures of the two groups varied significantly in the STRUCTURE cluster. The author believes that this may have been related to land use (QDDY was present in perennial agricultural land; however, QDY was present in newly cultivated land. As a result, QDDY were subjected to greater anthropogenic interference).

With the development of sequencing technology, a reduced-representation genome sequencing method with high-throughput single-nucleotide polymorphisms discovery was used for the genetic differentiation of earthworms (Marchán et al., 2020; Yuan et al., 2020). With the further availability of reference genomes, this method will be more useful for earthworm genetics.

## Conclusion

In summary, we developed microsatellite molecular markers and designed 12 pairs of corresponding polymorphic primers for *M. vulgaris*. The genetic diversity and population structures of five *M. vulgaris* populations were explored via mitochondrial COI genes and microsatellites. The genetic diversity was at a moderate level and the genetic structure revealed that the five populations could be divided into three genetic groups. *M. vulgaris* populations were not genetically isolated by distance at small scales, and different land use patterns will lead to genetic differences in population. The aim of the present study was to further inspire and facilitate intense research on *M. vulgaris* genetics.

## Data Availability Statement

The datasets presented in this study can be found in online repositories. The names of the repository/repositories and accession number(s) can be found below: NCBI GenBank, COI1 accession numbers: SUB9429448 Seq1 MW861684–SUB9429448 Seq10 MW861693; SSR accession numbers: BankIt2446728 Seq1 MW858330–BankIt2446728 Seq12 MW858341.

## Ethics Statement

Ethical review and approval was not required for the animal study because Earthworms are common soil animals and are not listed in IUCN Red List of Threatened Species.

## Author Contributions

HL: conception and design. NX: development of methodology. ZQ: acquisition of data. YF, NX, ZQ, and JC: analysis and interpretation of data. YF, HR, and HL: writing, review, and/or revision of the manuscript. All authors contributed to the article and approved the submitted version.

## Conflict of Interest

The authors declare that the research was conducted in the absence of any commercial or financial relationships that could be construed as a potential conflict of interest.

## Footnotes

1 http://taylor0.biology.ucla.edu/
